# Driver Anxiety Detection Based on Seated Pressure Characteristics and Identification of Anxiety-Inducing Scenarios

**DOI:** 10.3390/s26041162

**Published:** 2026-02-11

**Authors:** Xiaoyan Yang, Yi He, Zhiqiang Wen, Weiwei Wang, Mengmeng Gao

**Affiliations:** School of Design and Art, Shaanxi University of Science and Technology, Xi’an 710021, China

**Keywords:** seating pressure distribution, driver anxiety, driving scenarios, cosine similarity

## Abstract

Driving anxiety is a major issue that compromises the safety and experience of driving. It has been demonstrated that negative emotions with a high arousal factor like anxiety are manifested in body posture and sitting behavior. This paper will investigate one of the ways of identifying anxiety by assessing the pressure distribution in the sitting posture, and discuss driving situations that have a strong correlation with causing anxiety. Thirty people were recruited through a campus social media platform. The experimental design was a one-factor within-subject experimental design in which the researcher used standardized audio materials and a digital countdown task as a means (or inducement) of achieving calm (baseline) and anxiety, respectively. The induction effects were validated using the Self-Assessment Measure (SAM). Also, a pathway accommodating eight driving conditions was established to address the depth of pressure distribution in each condition by means of pressure mats to examine the behavior of the subjects in the relaxed and anxious conditions. The evaluation of both subjective and objective data was performed using the Wilcoxon signed-rank test, and at the same time, we explored the relationships that existed among the driving situations and anxiety levels. The research findings reveal the following: (1) Compared to baseline emotional state, anxiety-induced conditions exhibit heightened pressure distribution and increased volatility in the thigh, hip, and lower back regions, accompanied by greater anterior–posterior center-of-gravity sway. (2) The study identified 40 significant features distinguishing anxiety from calmness, including aTHR_Max and rCOPBTL_Std, primarily distributed across the left leg, right hip, and lower back regions. (3) Through baseline correction and cosine similarity analysis, scenarios prone to triggering anxiety were identified as those involving high uncertainty and high interactivity (e.g., traffic congestion and entering roundabouts); scenarios characterized by continuity and high predictability (e.g., consecutive turns and parking) showed weaker associations with anxiety. This study provides new data support and design rationale for in-vehicle emotion recognition systems and emotion-intervention-based human–machine interaction design.

## 1. Introduction

Traffic accidents are a major cause of casualties and property damage. The World Health Organization’s latest *Global Status Report on Road Safety 2023* reveals that traffic accidents claim approximately 1.2 million lives annually and injure millions more [1]. The number of serious injuries and fatalities resulting from accidents caused by negative emotions experienced by drivers during driving tasks has been rising year by year. Negative emotions can influence driving behavior by directly altering actions or indirectly distracting drivers, thereby increasing the risk of traffic accidents [2]. Anxiety is a common negative emotional state during driving that may lead to excessive caution or indecision, posing a threat to road safety [3]. Therefore, identifying drivers’ emotional states and studying which scenarios trigger negative emotions are crucial for reducing accident rates and enhancing the driving experience.

In recent years, emotion recognition technology has garnered significant attention in the field of intelligent driving. Intelligent driving assistance systems can detect the driver’s emotional state and dynamically adjust intervention strategies in real time, thereby enhancing driving safety and improving the driving experience [4]. Most existing research has focused on self-reports, facial expression recognition, and physiological-signal monitoring. However, these methods are constrained by limitations such as high subjectivity, accuracy susceptible to environmental interference, and significant invasiveness. Seat posture, as a critical indicator of driving behavior, carries substantial latent emotional data and can serve as an additional information source to support emotion recognition methods [5]. However, existing research has primarily focused on the impact of seating posture pressure on driving comfort, with limited exploration into how seating posture pressure distribution can reflect driving emotions.

### 1.1. Emotion Recognition Methods

A driver’s emotional state significantly impacts driving performance, particularly as negative emotions such as anxiety and anger may compromise driving safety. Research on emotion recognition primarily focuses on facial expressions, behavioral actions, speech, and physiological signals [3,6]. By integrating knowledge from computer science, artificial intelligence, and psychology, it aims to identify and classify an individual’s emotional state. Among these approaches, emotion recognition based on physiological signals is currently the most widely studied. Physiological signals (such as EEG and ECG) are directly linked to the autonomic nervous system, providing objective measures of intrinsic emotional responses that are less susceptible to subjective masking [7]. Healey, J. et al. [8] determined relative stress levels by collecting various physiological signals from drivers during actual driving tasks. The results indicated that skin conductance and heart rate metrics were most closely correlated with driver stress levels; Zhou, M. [9] proposed an emotion analysis model based on Long Short-Term Memory (LSTM) and electroencephalograms (EEGs), creating an EEG dataset specifically designed to evaluate product-related emotions. Hassn, M. et al. [10] primarily investigated the relationship between skin conductance signals, photoplethysmography, and electromyography signals and emotional stress. They extracted statistical features from multimodal signals and employed a modified Gaussian support vector machine (FGSVM) to achieve five-class classification. Facial expression-based visual analysis is an intuitive and readily accessible non-contact method for emotion recognition. Zheng et al. [11] proposed a DCNN-based FLF-TAWL network capable of adaptively capturing key facial regions to enhance the effectiveness of facial recognition.

Emotion recognition based on behavioral characteristics involves capturing facial expressions, head position, limb movements, and sitting posture through devices such as cameras and tactile sensors for classification and identification [12]. Like facial expressions, many postural responses are innate and exhibit cross-cultural universality [13]. Santhoshkumar, R. [14] proposed a deep learning-based method for recognizing emotions from human body movements, achieving efficient identification of emotional states by analyzing body motion features. In a review conducted by Noroozi et al. [15], a comprehensive framework for automatic emotion recognition from limb gestures was defined by introducing several emotion models and emotion recognition components.

Based on a review of the current state of emotion recognition research, methods for identifying negative emotions in drivers still face several challenges. For instance, physiological-signal-based monitoring approaches exhibit a degree of invasiveness, while image recognition efficiency is significantly affected by environmental factors.

### 1.2. Pressure Distribution Detection Technology

Pressure distribution detection technology utilizes pressure sensors to capture pressure distribution patterns on body contact surfaces. Compared to traditional emotion recognition methods, pressure sensors are non-invasive, have minimal impact on the driving experience, and can promptly detect emotion-driven, unconscious postural adjustments [16]. The current field of pressure sensor recognition primarily focuses on posture recognition and comfort recognition. Li, M. [17] investigated the effects of varying driving durations on pressure distribution and perceived comfort at the passenger–seat interface; Ma, C. [18] developed a system utilizing an intelligent cushion to assess user posture and body sway, identifying activities from information concealed within sitting postures. Qisong, H. [19] employed six flexible pressure sensors to classify seven distinct health-related sitting postures; Tsai [20] developed a sitting posture recognition system named SPRS, identifying key regions on the chair surface that capture fundamental posture characteristics and employing multiple machine learning techniques to recognize ten common sitting postures.

Research on driver emotion recognition using pressure distribution detection technology remains limited, though a small number of studies have explored the potential for identifying emotions through pressure changes. For example, Hidalgo-Muñoz [21] identified emotion-specific features from a large set of human motion descriptors and considered seated postures, enabling the proposed recognition system to achieve very high emotion recognition rates. Dzedzickis, A. [22] reviewed research on emotion recognition methods based on facial expressions, body postures, and gesture analysis, comparing them with other previous emotion recognition approaches and highlighting their future application prospects. Gravina, R. [5] designed a system based on body-worn inertial sensors and pressure detection modules, identifying four common emotion-related activities through feature-level fusion techniques. Yulia, S. [23] revealed correlations between pressure sensor readings and human emotional states through a hardware–software system measuring armchair posture. Consequently, while emotion recognition methods based on pressure cushions hold significant potential, they require further exploration and validation.

### 1.3. Emotions and Driving Scenarios

Driving emotions are not solely connected with autonomous driver traits; however, they play a highly important role in driving situations [24]. Research indicates that traffic congestion typically elevates drivers’ anxiety levels, and during peak hours, prolonged waiting and slow-moving traffic make drivers more prone to irritability and even anger [5].

Li, G. [25] found that drivers become more aggressive after experiencing traffic congestion, which has a cumulative negative impact on drivers’ emotional arousal. Ma Y [26] conducted video analysis of ride-hailing drivers’ facial expressions and behaviors under two conditions (with/without passengers) and three typical scenarios, the study revealed that negative emotions and aggressive driving behaviors occurred more frequently when passengers were absent; Xiaoming, T. [27] collected multimodal physiological data across five driving scenarios to analyze driving behavior, demonstrating correlations between physiological metrics and driving actions; a review of the literature by Ni, J. [28] indicated that road complexity and traffic congestion are critical.

Recent studies further confirm the significant role of different driving scenarios in emotional regulation. Arash, T. [29] monitored drivers’ heart rates and facial expressions, finding that large vehicles on the road were associated with the greatest increases in drivers’ heart rates and negative emotions; Hamad [30] employed data-driven unsupervised machine learning to classify driving behaviors at three roundabouts, investigating driving behaviors at roundabouts within mixed traffic environments.

Most current research on driving scenarios focuses on traditional emotional indicators such as heart rate, skin conductance, and facial expressions, while paying less attention to biomechanical signals during driving. Furthermore, existing studies primarily describe correlations between anxiety and individual scenarios, lacking in-depth analysis of the differential mechanisms underlying anxiety expression across different driving contexts.

### 1.4. Aim of the Research

This study explores anxiety-related emotional characteristics and patterns based on seated pressure distribution, objectively identifying driving scenarios prone to triggering anxiety through measured pressure data. The research aims to reveal the associative characteristics between anxiety and seated pressure distribution, identify high-anxiety-risk scenarios, and provide theoretical foundations and data support for in-vehicle emotion monitoring and active intervention systems, thereby enhancing driving safety and comfort.

## 2. Methods

### 2.1. Participants

Participants were recruited through social media announcements within the university community. They were required to hold a valid driver’s license, possess at least one year of driving experience, and have a monthly driving mileage of 320–450 km (M = 385 ± 112). The study recruited 30 healthy drivers (16 males, 14 females) aged between 19 and 30 years (SD = 2.70). Participants were required to be in good physical health, possess normal vision or vision corrected to normal, and be free from psychological disorders, emotional disturbances, or neurological conditions. Each participant completed a health assessment and signed an informed consent form prior to the experiment.

### 2.2. Equipment

**Figure 1:**
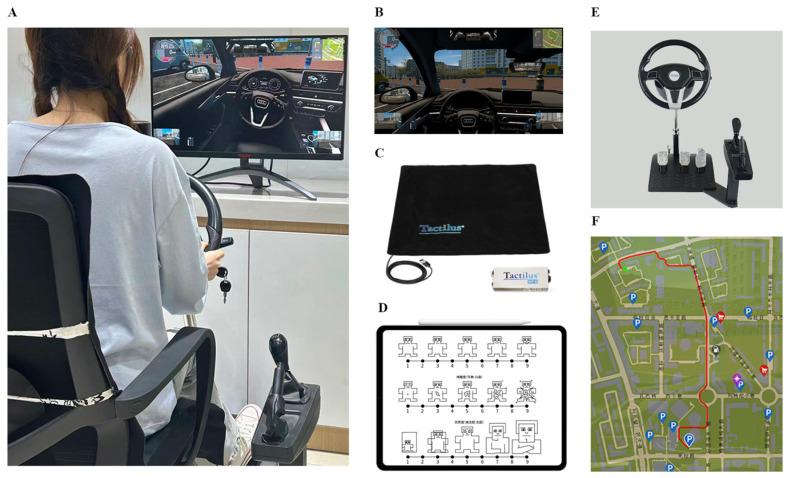
Experimental environment. (**A**) Experimental setup. (**B**) Driving scenario configuration. (**C**) Pressure data collection. (**D**) Subjective data collection. (**E**) Driving simulator. (**F**) Experimental route.

The experiment was conducted in a quiet space measuring 4 m × 4 m, with room temperature maintained at 25 °C. Adequate ventilation and lighting were provided to ensure subject comfort. As shown in Figure 1, the experimental setup consisted of a driving simulator with a fixed base, a display system, and a body pressure data acquisition system. The simulator was equipped with a steering wheel, accelerator, clutch, and brake pedals, complemented by a gearshift lever and handbrake, to replicate the vehicle’s operational mechanics and ergonomic layout. For data acquisition, two slim Tactilus pressure pads were installed on the seat cushion and backrest of the experimental chair with wrinkle-free surfaces to capture body pressure distribution in real time. The entire simulation environment was powered by 3D Instructor 2.0 software, displaying the vehicle’s dashboard, rearview mirror, and side mirrors on both sides to provide participants with a more realistic driving experience.

### 2.3. Experimental Design

#### 2.3.1. Emotion Induction

The experiment employed a single-factor within-subjects design, with emotional state (anxiety, calmness) as the sole independent variable. Anxiety was induced using the “digit backward counting task” previously employed by Keogh [31]. Participants were instructed to count backward from 1000 in increments of 3 for two minutes. Prior to the task, subjects were informed that this was an intelligence-related test where their performance would be compared to that of other participants. If anxiety was induced, subjects were to signal the experimenter by pressing a buzzer, with the buzzer press time recorded as the onset of anxiety. To induce emotional calmness, we employed video-based induction techniques, selecting non-narrative videos featuring landscapes, scenery, or natural settings as calming stimuli. Each induction video was required to last two minutes to ensure subjects’ emotions were sufficiently induced while avoiding “emotional fatigue” caused by prolonged exposure.

During the emotional induction process, real-time body pressure data was collected using the Tactilus pressure cushion. Following the induction, participants completed the SAM questionnaire to assess whether their emotional state had undergone significant changes, as shown in Figure 1D.

To validate the induction effects of the emotion-induction experiment, a pre-experiment was conducted with 5 participants (3 males, 2 females; mean age = 25.6 years, SD = 2.1 years) prior to the formal experiment. The results indicated that compared to the calm condition, participants exhibited significantly lower valence ratings (t (4) = 5.32, *p* < 0.01) and significantly higher arousal ratings (t (4) = 6.18, *p* < 0.01) following anxiety induction. This confirms the method’s ability to successfully induce distinct emotional states.

#### 2.3.2. Driving Scenarios

The simulated driving environment was set for urban driving, featuring a 1.5 km road segment. Drivers were required to navigate to a designated location at a speed limit of 40 km per hour. The simulated conditions were daytime, clear weather, and high visibility. Participants were asked to drive for approximately 10 min in accordance with traffic rules. They were free to drive according to their own habits, with no scoring requirements. The driving route and experimental scenario are shown in Figure 1F.

Prior to the experiment, a questionnaire assessing anxiety-inducing driving scenarios was designed and distributed. This questionnaire compiled 20 complex or potentially stressful situations commonly encountered in urban driving. Respondents were asked to rate the potential anxiety level of each scenario using a five-point Likert scale (1 = “Not anxious at all,” 5 = “Extremely anxious”).

**Figure 2:**
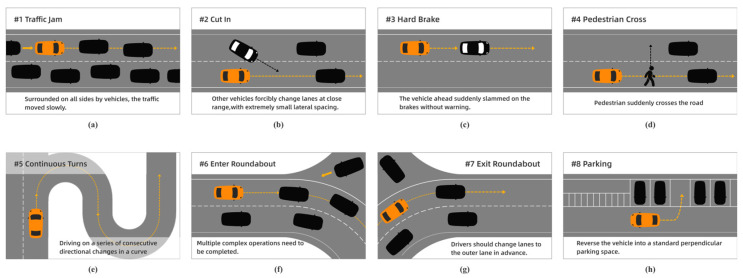
Experimental scenario diagram. (**a**) Congested traffic, (**b**) lane change, (**c**) emergency braking, (**d**) pedestrian crossing, (**e**) consecutive turns, (**f**) entering a roundabout, (**g**) exiting a roundabout, (**h**) parking.

Based on statistical analysis of the valid questionnaires collected (*n* = 60), the mean anxiety score and standard deviation were calculated for each scenario. As shown in Figure 2, the eight scenarios with the highest mean anxiety scores and the smallest standard deviations were ultimately selected as the experimental scenarios for this study.

### 2.4. Procedure

Step 1: Pre-test Questionnaire. Participants provided informed consent prior to participation and answered questions regarding traffic regulations and driving behavior.

Step 2: Driving Training. To familiarize participants with the simulator controls, a 10 min training session was conducted prior to the experiment, during which no actual driving scenarios or routes were revealed. Under the guidance of the experimenter, participants practiced basic operations such as using the steering wheel, accelerator, brake, and gearshift controls, as well as the navigation system.

Step 3: Calmness Induction and Driving. Subjects were shown a relaxing video to induce a calm state, during which their stress distribution data was recorded and they completed the SAM questionnaire. Subsequently, subjects began simulated driving while maintaining this calm state. To sustain this state, subjects wore headphones continuously listening to calming music (*Arthur’s Nocturne*). The driving task involved a route from Location A to Location B, comprising eight scenarios, as depicted in Figure 2. Timestamps and somatic pressure data were recorded for each scenario. After completing the driving task, participants completed the SAM questionnaire to assess their emotional state.

Step 4: Anxiety Induction and Driving. After a brief rest period, subjects underwent anxiety induction using the method described in Section 2.2. Their body pressure data were recorded in real time throughout the induction. Following completion, subjects complete the SAM questionnaire to assess the effectiveness of the induction. Next, subjects were instructed to simulate driving while in an anxious state, wearing headphones that played anxiety-inducing music (*Ligeti’s Requiem*) to maintain this state [32,33]. Similarly, we recorded the time points at which each scenario occurred along with body pressure data, and participants completed the SAM questionnaire after driving.

**Figure 3:**
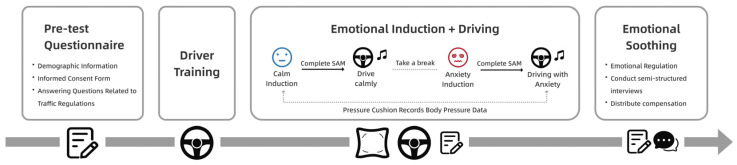
Experimental flowchart.

Step 5: Emotional Regulation and Interview. After the experiment concluded, participants still experiencing high levels of anxiety underwent emotional regulation through methods such as watching videos. A semi-structured interview was then conducted with participants regarding the experimental process, driving experience, and changes in anxiety levels. All qualified participants received appropriate compensation. The overall process is shown in Figure 3.

### 2.5. Data Analysis

#### 2.5.1. Analysis of Self-Reported Data

**Figure 4:**
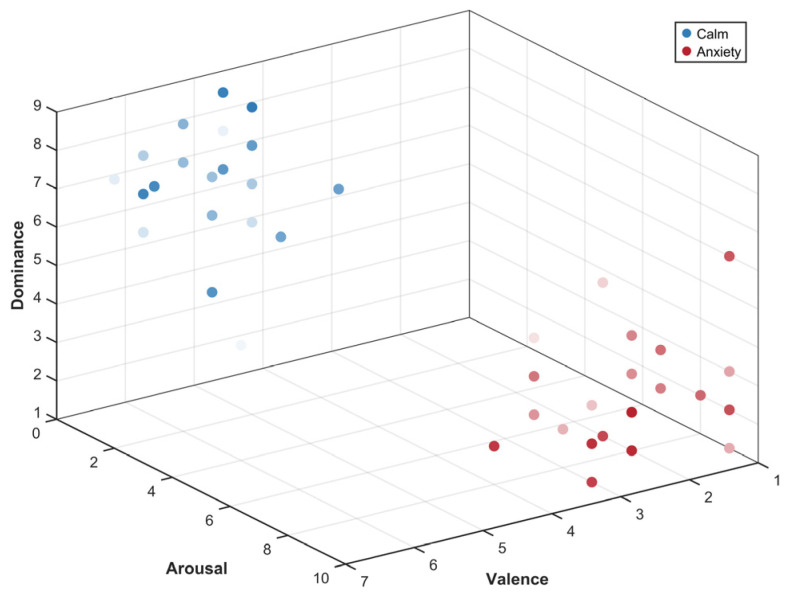
Scatter plot of SAM results.

A normality test was conducted on the self-reported data, yielding a significance *p*-value of 0.001 (*p* < 0.05). The null hypothesis was therefore rejected, indicating that the sample data did not follow a normal distribution. The Wilcoxon signed-rank test was conducted across three dimensions: valence, arousal, and dominance. Significant differences were confirmed for all three scores at *p* < 0.001, validating the effectiveness of emotion induction. Given the non-normal distribution of the sample, the effectiveness of emotional regulation was assessed by examining the median and interquartile range. As shown in Table 1, calm and anxious emotions were successfully induced. Figure 4 displays the distribution of all participants’ self-reported SAM scores across the three-dimensional affective space (valence–arousal–dominance) under both emotional conditions. Each point represents a subject’s subjective rating under the corresponding condition, with blue tones indicating the calm condition and red tones indicating the anxiety condition. Overall, the red and blue clusters show almost no overlap in three-dimensional space, demonstrating the effectiveness of the emotion induction employed in this study.

#### 2.5.2. Pressure Data Processing

The Tactilus pressure measurement system in the experiment registered and gathered stress measurements with emotional induction and driving. The pressure cushion is made up of a 32 × 32 sensor array with a 5Hz sampling frequency.

**Figure 5:**
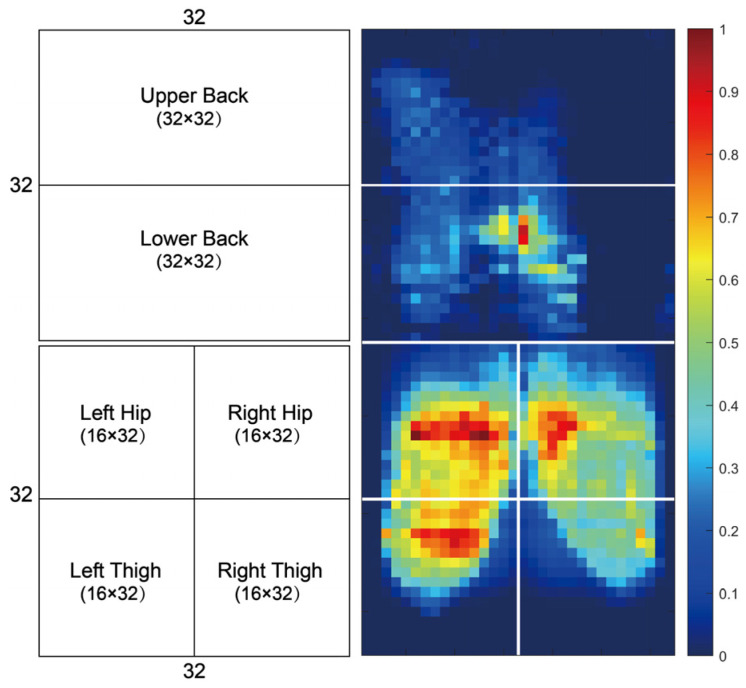
Body pressure distribution heatmap.

The first step was to process the pressure data to exclude outliers and maintain the accuracy of the data. We processed the obtained data with MATLAB (R2024b) software. We then reconstructed each frame vector, subdividing the pressure pad on the seat into six great areas as indicated in Figure 5: left buttock (BTL), right buttock (BTR), left thigh (THL), right thigh (THR), upper back (UB) and lower back (LB). We determined the average pressure (mmHg), sensing area (non-zero points), and pressure center point (row col), as well as the area ratio of each to the total area of each region. A set of 36 variables of pressure values was obtained, as demonstrated in Table 2. Twelve pressure variables relate to contact pressure, twelve to contact area, and twelve reflect the geometric center position of the force-bearing region. This converts the high-dimensional distribution map of pressure variations into quantitative feature vectors that are capable of indicating the differences in body posture when one is in an anxious or a calm condition.

#### 2.5.3. Time Annotations

Emotion-related physiological and behavioral responses typically develop gradually over a few seconds and persist, rather than occurring instantaneously. Multiple studies on emotions and driving behavior employ a 3–10 s time window to characterize changes before and after emotion induction. This duration effectively captures both the emergence and intensification phases of emotions while avoiding the inclusion of irrelevant behaviors or state drift associated with longer windows [34,35]. Therefore, in this study, the moment when a subject honked the horn was marked as the most prominent instance of that emotion. Data from 5 s before and after this moment were extracted: the preceding 5 s represented the emergence process of the emotion, while the following 5 s represented its ongoing development, totaling 50 frames of data. 

We calculated the stress variables for each emotion, forming a 36 × 50 emotional feature set [8,36]. For continuous events such as sustained turning or parking, we directly captured the first 10 s after the event began to assess the state or load during the action maintenance phase. Regarding roundabouts, studies indicate that approaching and entering a roundabout induce higher anxiety than driving within it [37]. Thus, data centered around roundabout entry/exit times, with 5 s before and after each event, were extracted. Similarly to the emotional feature set, a 36 × 50 feature set was computed for each scene under both anxious and calm states for subsequent comparison.

#### 2.5.4. Dynamic Baseline Calibration

The anxiety-state feature table established during the stationary induction phase was used for comparison with driving-scenario data to identify scenarios whose pressure distribution patterns are similar to those observed under stationary anxiety, and thus are more likely to elicit driver anxiety. However, since driving actions themselves (such as braking or steering) cause changes in body pressure distribution, direct comparison may lead to misinterpretation of these action differences as emotional effects. Therefore, this study employs calm-driving data as the baseline for dynamic baseline correction in each scenario, thereby eliminating interference from action factors.

The calibrated feature difference vectors are then subjected to cosine similarity calculations with the feature difference vectors derived from anxiety and calmness under static induction. Cosine similarity focuses on the directional consistency between multidimensional feature vectors, effectively reflecting the relative structural characteristics of pressure distribution across regions while remaining unaffected by overall amplitude variations. This property grants it enhanced robustness in analyzing body pressure and physiological signals, where significant inter-individual differences exist. The specific methodology is as follows:

Step 1: Construction of Anxiety–Calmness Difference Vectors Under Static Conditions

In the static induction experiment, multiple statistical features are computed across six body regions (hip, thigh, back, etc.) and concatenated into pressure feature vectors. Feature vectors for the anxious and calm states are denoted as $F_{\mathrm{static}}^{\mathrm{anx}}$ and $F_{\mathrm{static}}^{\mathrm{calm}}$. The anxiety prototypical difference vector is defined as:

(1) $$\Delta_{static}=F_{static}^{anx}-F_{static}^{calm}$$

This vector describes the typical pattern of stress distribution changes that occur when anxiety replaces calmness.

Step 2: Construction of Differential Vectors in Driving Scenarios

For each driving scenario *i*, we obtain the stress feature vectors $F_{\mathrm{scene},i}^{\mathrm{anx}}$ and $F_{\mathrm{scene},i}^{\mathrm{calm}}$, representing anxious and calm driving, respectively. The scenario difference vector is defined as:

(2) $$\Delta_{\mathrm{scene},i}=F_{\mathrm{scene},i}^{\mathrm{anx}}-F_{\mathrm{scene},i}^{\mathrm{calm}}$$

This step eliminates the stress variations inherent to the driving task itself under calm conditions, retaining only the additional effects induced by anxiety.

Step 3: Similarity Metric

To assess the consistency between anxiety-related changes in a specific scenario and the typical pattern of static anxiety, cosine similarity is employed:

(3) $${\mathrm{Sim}}_{i}=\frac{\Delta_{\mathrm{scene},i}\cdot \Delta_{\mathrm{static}}}{∥\Delta_{\mathrm{scene},i}{∥}_{2}\;∥\Delta_{\mathrm{static}}{∥}_{2}}$$

Here, ⋅ denotes the vector dot product, and $∥\cdot {∥}_{2}$ 2 denotes the L_2_ norm. The closer the ${\mathrm{Sim}}_{i}$ value is to 1, the more the stress distribution pattern induced by the scenario resembles anxiety characteristics, indicating a higher likelihood of triggering anxiety.

## 3. Results

### 3.1. Pressure Variables

**Figure 6:**
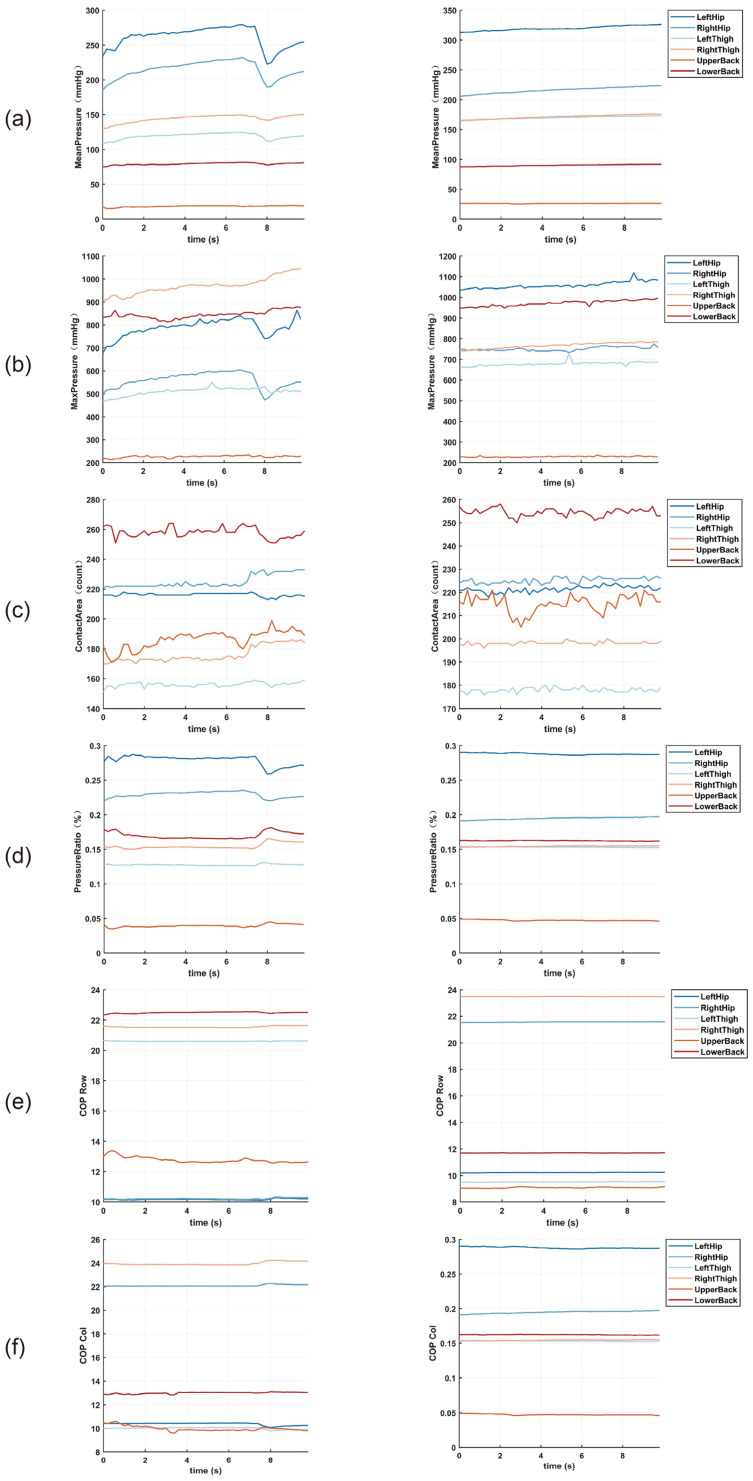
Changes in pressure parameters for the 95th percentile subject. Left panel: anxious state; right panel: calm state. (**a**) Mean pressure, (**b**) maximum pressure, (**c**) contact area, (**d**) regional pressure ratio, (**e**) COP row, (**f**) COP col.

A consistent trend was observed in the stress parameters of the 25 subjects. Figure 6 shows the variation in the stress parameters of the subject in the 95th percentile. Under calm conditions, average pressure and other measures in all the regions had a slight, smooth upward tendency over time, and no observable variation or sudden reaction occurred; under the state of anxiety, all four parameters (average pressure, contact area, peak pressure, and ratio of pressures) in lower-body weight-bearing areas, including the left thigh/right thigh and left hip/right hip, and the lower back region, increased by around 10–20 percent in value, compared to the state of calm. The left hip and right hip had the largest variations in the metrics of pressure, with a maximum range of 30–40 mmHg, whereas the upper back was low in general and had weak variability, suggesting that the upper back is the least loaded during emotional transitions.

COP Row (anterior–posterior direction) also displayed stronger slow drift and periodic regression fluctuations (±0.51 unit) in anxiety, but in calm states, it exhibited a mostly straight curve. COP_Col (lateral direction) demonstrated a similar trend between both states with the fluctuations within the 1-unit range, which suggested the occurrence of emotional changes, which are mainly in the form of slight adjustments in the center of gravity along the anterior–posterior axis, but the balance control along the lateral axis remains consistent.

During anxiety, pressure indicators not only exhibit higher baseline values but also display sudden peak-to-trough fluctuations, corresponding to subjects’ unconscious postural adjustments or shifts in center of gravity. The simultaneous change in pressure ratio and COP_Row indicates that the subjects tend to increase the use of lower-body compensations (higher pressure ratio) and anterior–posterior sway (COP_Row changes) to stabilize sitting positions when they sense the presence of psychological tensions; thus, they tend to cope with the psychological tension.

Overall, anxiety is most prominently reflected in increased pressure distribution and heightened fluctuations in the lower limbs (thighs, hips) and lower back region, along with greater sway in the anterior–posterior direction; the upper back and lateral balance remain largely unaffected. These spatiotemporal dynamic characteristics provide key, quantifiable biomechanical markers for subsequent real-time anxiety detection algorithms based on seated pressure and center of pressure (COP).

### 3.2. Analysis of Features

**Figure 7:**
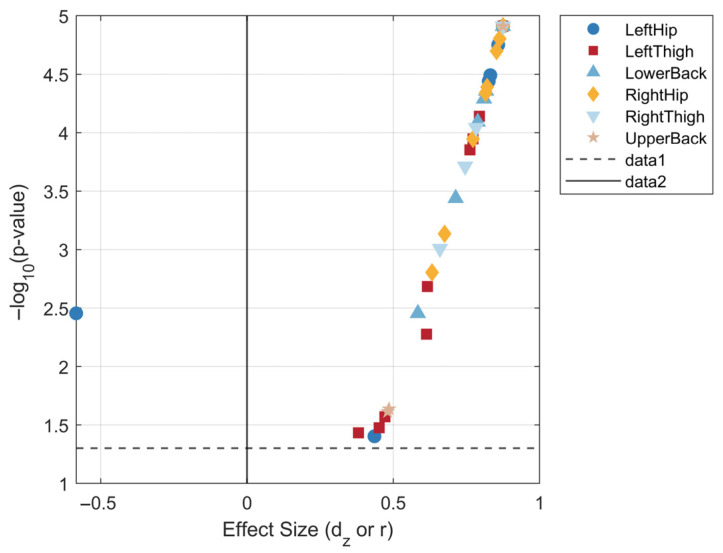
Effect size volcano plot of features.

From twenty-five subjects, we obtained 50 frames × 36 temporal features each under both anxious and calm emotional states. After preprocessing, statistical features were extracted: mean, standard deviation, and maximum value. We first calculated the “anxiety–calmness” pairings for each extracted statistical feature $Dj,i=X_{i,j}^{Anx}-X_{i,j}^{Calm}$ (i = 1…25 subjects, j = 1…F features). The Lilliefors test was used to determine Dj whether data followed a normal distribution (significance level α = 0.05). If normality was not rejected (h = 0), a paired *t*-test was performed to compare mean differences between the two emotional states; otherwise, the Wilcoxon signed-rank test was used to compare median differences. Both methods provided *p*-values describing statistical significance, with the *p*-value threshold set at 0.005. Based on the measured *p*-values, any feature failing the significance test was automatically discarded. The remaining features were ranked according to their correlation strength, yielding a total of 40 significant features. Figure 7 presents a volcano plot of feature effect sizes, enabling an intuitive comparison of feature significance and magnitude.

**Figure 8:**
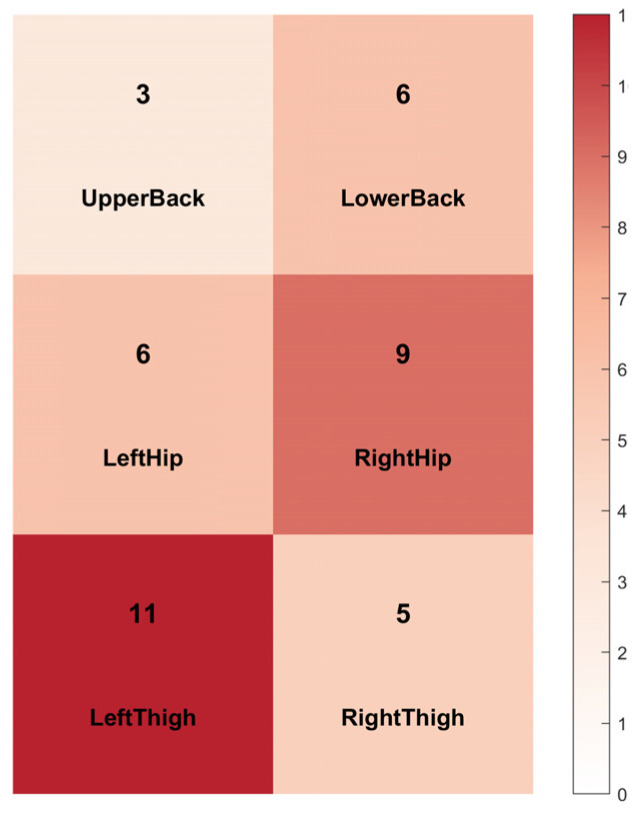
Effective features of each area.

An important reason why feature analysis was conducted was to obtain a better understanding of human emotions and bodily movements. The effective features by region can be observed in Figure 8, where 11 major features are seen in the left leg (mean pressure ratio and maximum column value of COP), with the biggest effect size being 0.8745. This indicates that pressure and pressure center displacement in the thigh region are most sensitive to anxiety. There are nine distinct features of the right buttock, and the indicators based on the COP row and ratios are very prominent. Among the 40 features capable of significantly distinguishing anxiety from calm states, the lower back, left thigh, and right hip regions collectively contribute 64.1% of the significant features. This implies that the subjects mainly adjust to psychological stress during anxiety with force distribution and center-of-gravity changes in the lower back and lower limbs (particularly of the left thigh and right hip).

For features significant in *t*-tests, we calculated the paired Cohen’s d; for features significant in Wilcoxon tests, we calculated the rank-biserial correlation coefficient r. The top ten features that were significant in paired tests, along with their effect sizes, are recorded in Table 3. The thigh-related indicators constitute 4/10 of them, including the maximum values and the change metrics; in the state of anxiety, the thigh region experiences more peak loading but more severe tremors. The hip region accounts for 3 out of 10, including the standard deviation of COP longitudinal displacement and pressure ratio/contact area variability, and it demonstrates more significant micro-adjustments of the hip center of gravity during the state of anxiety. There is a single feature that includes the lower and the upper back: mean pressure in the lower back and mean COP column in the upper back were introduced as secondary but rather important distinguishing features. This implies that the back is not as sensitive as the lower limbs, but at the same time, its load-bearing ability is also heightened in the state of anxiety.

### 3.3. Similarity Calculation and Scene Classification

**Figure 9:**
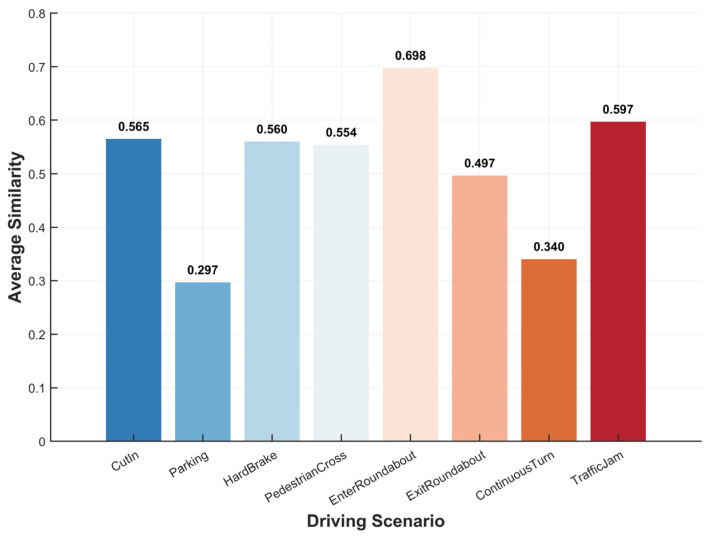
Comparison of average similarity across driving scenarios.

Due to missing body pressure data and time stamp desynchronization in certain scenarios for some subjects, the final dataset comprised body pressure features collected from 18 subjects across eight typical driving scenarios. These were compared to pressure difference vectors under static anxiety-inducing conditions after baseline correction was carried out, as shown in Section 3.3, and then the cosine similarity was computed for each scenario. The findings are presented in Figure 9, which shows that traffic congestion (0.597) and entering roundabouts (0.698) have the largest similarity, which means that the majority of the participants are more likely to experience a state of anxiety, which happens because of stasis in these situations of conflict or congestion. Other maneuvers, such as cutting off another vehicle (0.565), emergency braking (0.560), and pedestrians crossing (0.554), also demonstrate moderate similarity, and this means that there are strong correlations among these abrupt or urgent maneuvers during driving activities and anxiety. The factor with the next lowest value is continuous turning (0.340), which implies that the likelihood of anxiety being aroused is lower in the case of regular but individual operations. Parking (0.297) was the least similar and exhibited the most variation with regard to the subjects, and was less tied to the emotion and more dependent on the individual’s experience during the operation.

Overall, scenarios involving vehicle control generally evoke more uniform cognitive responses, whereas those involving abrupt events and intricate maneuvers—such as parking or successive turns—exhibit greater individual variability. This discovery offers valuable insights into the fundamental mechanisms of driving anxiety, indicating that particular types of driving situations may necessitate more tailored intervention approaches.

**Figure 10:**
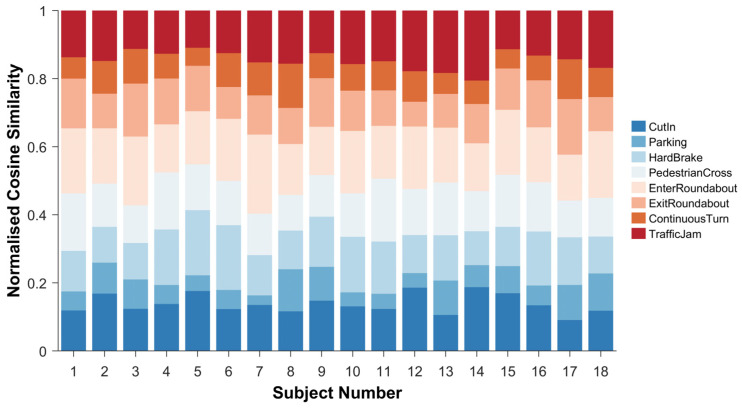
Cosine similarity stack plot for each scene by subject.

The stacked plots in Figure 10 reveal distinct individual differences in similarity distribution across participants. Some subjects (e.g., Subjects 2, 7, and 15) maintained consistently high similarity across all scenarios, potentially indicating a stable cognitive style. In contrast, others (e.g., Subjects 5, 12, and 17) exhibited greater variability between scenarios, suggesting that their cognitive processing is more context-dependent. Driving scenarios involving sustained vehicle control (e.g., traffic congestion, roundabouts) tended to elicit more consistent physical response patterns, whereas scenarios involving sudden events or complex maneuvers (e.g., consecutive turns, parking) exhibited significant individual differences. This pattern of individual variation suggests that driving experience and skill confidence may serve as potential moderating factors influencing the stability of anxiety responses. Qualitative analysis incorporating participants’ driving background information reveals that relatively stable similarity distributions are more common among subjects with greater driving experience or higher self-assessed skill levels. Conversely, subjects exhibiting greater fluctuations in similarity often display novice characteristics or anxiety responses that are more sensitive to specific situations. This finding provides empirical evidence for understanding the mechanisms underlying the development of driving anxiety.

## 4. Discussion

### 4.1. Biomechanical Characteristics of Anxiety in Terms of Sitting Posture Stress

This study found that compared to a calm state, anxiety primarily manifested as a general increase in pressure levels in the lower limbs and lower back, enhanced temporal fluctuations, and a greater amplitude of pressure center oscillation (COP_Row). A total of 40 features significantly distinguishing anxiety from calmness were identified, with those from the left thigh, right buttock, and lower back accounting for 64.1% (Table 4). This indicates that individuals primarily regulate psychological stress during anxiety through adjustments in force distribution across the lower limbs and lower back, along with subtle shifts in the center of gravity. These findings align with Süß’s, F. [38] research on trunk muscle tension patterns under mental stress, supporting the validity of seated pressure characteristics as anxiety indicators. Notably, the left thigh and right hip exhibited the highest sensitivity. This may relate to drivers’ behavioral tendency to unconsciously tense the left leg and shift weight to the right hip to maintain support during anxiety.

Anxiety is a typical state of tension and defensive readiness that induces generalized muscle activation and, in turn, alters sitting load distribution and contact area. In anxious states, increased activation of core and lower limb muscles is required to maintain postural stability, which tends to concentrate pressure in the lower limbs and lower back and leads to more pronounced temporal fluctuations [39]. Emotional arousal also affects postural control strategies, as individuals adjust their balance behavior to preserve stability or a sense of safety, which is reflected as increased anterior–posterior COP fluctuations (COP_Row). One plausible explanation is that, from an evolutionary perspective, threats were more likely to arise from the front or rear, resulting in anxiety-related bodily preparedness that is mainly expressed along the sagittal plane. Consequently, the increased anterior–posterior sway may represent a bodily manifestation of a subtle and repetitive “approach–avoidance” conflict [40], in which individuals unconsciously lean forward to prepare for action and backward to prepare for withdrawal, thereby enlarging the COP trajectory in this direction. Conversely, lateral balance primarily relies on automated neural control systems to maintain equilibrium, being less susceptible to emotional and cognitive interference [41]. This explains why lateral balance remains stable during states of anxiety.

The present study illustrates that abstract emotional states can be turned into a set of quantifiable biomarkers, which are directly associated with biomechanical responses. Compared to commonly used signals such as EDA, heart rate, or facial expressions, seated pressure reflects the driver’s unconscious postural and load-adjustment behaviors during states of anxiety. It offers the advantages of high concealment, difficulty in feigning, and ease of in-vehicle integration. On both temporal scales and information levels, it can serve as a complementary signal to existing modalities.

### 4.2. Anxiety-Inducing Scenario Mining Based on Pressure Pattern Similarity

The similarity analysis of situations in this study suggests that the most similar situations are those where there is traffic congestion and where drivers are entering a roundabout, implying that these situations pose the greatest chances of arousing patterns of anxiety comparable to those induced by a stationary condition. This result is in line with the literature on road complexity and driver mental load: intersections, roundabouts, and congestion situations increase information uncertainty and multi-agent interaction pressure, thus augmenting physiological and subjective stress reactions [28]. Conversely, there are also moderately high similarity rates in brief yet unpredictable events (like vehicles swerving, emergency braking, or people crossing), which means that they can also cause an acute response to stress within an incredibly short period of time. The results of empirical research indicate that in relation to abrupt hazards, drivers experience considerable acute heart rate, respiration, and even skin conductance increments. This acute, extreme physiological process has a close relationship with the warning signs of emotional anxiety [42].

The similarity of sequential turns is at the low-to-medium level. One reason for this is that consecutive turns can be explained as a maneuver that is coherent yet predictable. Under these conditions, divers create movement plans and rhythmic control, which is the main tool used by drivers, and leads to comparatively small task uncertainty and cognitive load. Therefore, there is a smaller chance that this situation will cause major anxiety patterns. According to a study conducted by Lee on the mental workload of drivers operating advanced driver assist systems [43], task predictability is an important factor behind eliminating physiological driving stresses. Parking tasks had the greatest dissimilarity and the most variability among subjects, implying that mental responses due to parking are greatly reliant on individual skill, experience, and situation confidence, including bystander availability, the size of available parking areas, and traffic movement.

From a cognitive–physiological perspective, the above-mentioned pattern can be attributed to the following chain of congestion/roundabouts: multiple participants, highly dense decision points, and the need to continuously observe the behavior of other people; thus, cognitive load and emotional vigilance were continuously increased, and the rhythmic/predictable feature of unceasing turns eliminated, preventing the enhancement of emotional disturbances. These findings not only enhance our comprehension of driving emotion-generation mechanisms, but also give a theoretical premise for monitoring driver anxiety and individual intervention schemes that depend on postural stress and physiological attributes.

### 4.3. Shortcomings and Limitations

This study has yielded some meaningful findings regarding the identification of driver anxiety characteristics and scenario mining based on seated pressure. However, several limitations remain.

First, the sample size and representativeness are limited. The study population primarily consisted of young drivers and learner drivers aged 19–30, exhibiting limited demographic diversity. This sample characteristic partially restricts the generalizability of the findings to older drivers, professional drivers, or individuals with high trait anxiety. Drivers differing in age, driving experience, and risk sensitivity may exhibit distinct biomechanical response patterns under conditions of anxiety.

Secondly, the authenticity of the experimental environment requires improvement. In this study, anxiety states were primarily induced and measured within a simulated driving environment, which differs from the anxiety experienced during actual road driving. Simulated anxiety may lack the continuous risk perception, environmental uncertainty, and cumulative stress effects present in real traffic scenarios. Although driving simulators offer excellent experimental controllability and reproducibility, these differences may somewhat affect the ecological validity of the research findings.

Finally, with regard to controlling for individual differences and confounding factors, this research did not systematically account for individual factors potentially influencing sitting posture, such as height, weight, body type, long-term driving habits, or even clothing worn on the day of testing. These factors may act as confounding variables, affecting the universality and robustness of anxiety trait recognition.

To address these limitations, future research will expand sample sources to encompass a broader range of drivers and explore domain-specific modeling strategies tailored to different driving groups, thereby enhancing the method’s universality and robustness. By deploying pressure-sensing seat systems in real vehicles and conducting long-term natural-driving data collection, we will validate the stability and applicability of anxiety-stress patterns identified under laboratory conditions in real traffic environments, bridging the ecological gap between experimental research and practical application. Furthermore, incorporating upper-limb metrics such as steering wheel grip force or electromyography signals will establish a more comprehensive anxiety-sensing framework, enhancing interpretability across diverse driving behavior scenarios.

## 5. Conclusions

In general, this study proposes and validates a method based on seated pressure distribution characteristics and pattern exploration, further identifying driving scenarios highly correlated with anxiety. Among the most crucial ones are the following: (1) Seated pressure distribution as an effective biomarker of driver anxiety—The authors demonstrate the possibility of this distribution with the induction of high levels of anxiety in a driver, and these results are related to the characteristic scenarios of driving. (2) The dynamic correction of the baseline as an innovative tool—This method effectively removes the impact of driving operations on sitting posture and extracts emotion-related variations in pressure distribution within the analyzed sample. (3) Identification of key driving scenarios that induce high anxiety. Collectively, this work extends driving emotion recognition beyond facial expressions, speech, and invasive physiological signals to biomechanical behavioral signals that are more implicit, less controllable, and easier to integrate into intelligent cockpit systems, thereby enriching the methodological framework for driving emotion research. Furthermore, the findings provide direct design guidance and data support for developing in-vehicle real-time emotion monitoring and intervention systems based on intelligent seat cushions.

## Figures and Tables

**Table 1:** Nonparametric tests for emotions.

Emotional State	Dimensions	Median (Quartile)	*p*
Calm	Pleasure	5 (5~6)	*p* < 0.001
	Arousal	2 (2~3)	
	Dominance	8 (7~8)	
Anxiety	Pleasure	2 (1~3)	*p* < 0.001
	Arousal	8 (8~9)	
	Dominance	2 (2~3)	

**Table 2:** Feature set for binary emotion recognition under stress.

Category	Variable Name and Description		
Contact pressure (12)	Average contact pressure (mmHg)	Left (right) buttock:	avgBTL (avgBTR)
		Left (right) thigh:	avgTHL (avgTHR)
		Upper (lower) back:	avgUB (avgLB)
	Average contact pressure ratio	avgSUM:	(avgBTL + avgBTR +avgTHL + avgTHR + avgUB + avgLB)
		avgBTL/avgSUM, avgBTR/avgSUM, avgTHL/avgSUM, avgTHR/avgSUM, avgUB/avgSUM, avgLB/avgSUM	
Contact area (12)	Average contact area (count)	Left (right) buttock:	aBTL (aBTR)
		Left (right) thigh:	aTHL (aTHR)
		Upper (lower) back:	aUB (aLB)
		avgSUM:	aBTL + aBTR + aTHL +aTHR + aUB + aLB
	Average contact area ratio	aBTL/aSUM, aBTR/aSUM, aTHL/aSUM, aTHR/aSUM, aUB/aSUM, aLB/aSUM	
Center of pressure (12)	Row direction coordinate	Left (right) buttock:	rCOPBTL (rCOPBTR)
		Left (right) thigh:	rCOPTHL (rCOPTHR)
		Upper (lower) back:	rCOPUB (rCOPLB)
	Column direction coordinate	Left (right) buttock:	cCOPBTL (cCOPBTR)
		Left (right) thigh:	cCOPTHL (cCOPTHR)
		Upper (lower) back:	cCOPUB (cCOPLB)

**Table 3:** Top ten features and effect sizes.

Region	Feature Name	*p*-Value	Effect Size
Upper back	cCOPUB_Mean	1.23 × 10^−5^	0.875
Lower back	avgLB_Mean	8.76 × 10^−5^	0.681
Left buttock	rCOPBTL_Std	2.13 × 10^−4^	0.587
Right buttock	aBTR_Std	5.67 × 10^−5^	0.754
	avgBTR/avgSUM_Std	4.32 × 10^−4^	0.534
Left thigh	aTHL/aSUM_Max	9.81 × 10^−5^	0.698
	avgTHL/avgSUM_Std	3.28 × 10^−5^	0.742
	aTHL_Max	3.91 × 10^−4^	0.552
Right thigh	aTHR_Std	6.54 × 10^−5^	0.712
	avgTHR/avgSUM_Max	8.76 × 10^−5^	0.681

**Table 4:** Summary of anxiety-sensitive sitting pressure regions and key features.

Region	Quantity	Representative Characteristics
Left thigh	11	aTHL/aSUM_Max avgTHL/avgSUM_Std
Right buttock	9	aBTR_Std rCOPBTR_Max
Lower back	6	avgLB_Mean cCOPLB_Max

## Data Availability

The original contributions presented in this study are included in the article. Further inquiries can be directed to the corresponding author.
